# Enfermera comunitaria escolar e innovación docente para enseñar reanimación cardiopulmonar en la escuela a través de una *Flipped Classroom*

**DOI:** 10.1016/j.aprim.2023.102654

**Published:** 2023-05-16

**Authors:** Miriam Mendoza López, María Trinidad Pérez Rubio, Carlos Truque Díaz, Manuel Pardo Ríos

**Affiliations:** a UCAM Universidad Católica de Murcia, Murcia, España; b Consejería de Educación de la Región Murcia, Murcia, España; c Gerencia de Urgencias y Emergencias 061 de la Región de Murcia, Murcia, España

Integrar la educación para la salud en la escuela es fundamental ya que es una manera efectiva de promover y educar en hábitos saludables a los niños y jóvenes en su etapa de formación. La enfermera comunitaria escolar ofrece servicios de atención y cuidados de salud en las propias escuelas. Esta profesión desarrolla sus conocimientos en el punto donde convergen la atención de la salud y la educación, y desempeña un papel elemental en la promoción de la salud y el bienestar en el entorno escolar^1^.

La parada cardiorrespiratoria es la causa más importante de muerte por enfermedad cardiovascular y, por ello, el European Resuscitation Council (ERC) recomienda la formación poblacional en reanimación cardiopulmonar (RCP)^2^. En la mayoría de las ocasiones no se realiza una RCP, ni desfibrilación precoz, porque la mayoría de paradas cardiorrespiratorias suceden fuera del hospital y los ciudadanos no se sienten preparados para realizar las maniobras^3^, lo que lleva a cuestionarse si es posible pedir a un ciudadano que inicie estas maniobras sin haber recibido una formación básica.

Como consecuencia, las diferentes sociedades científicas están promoviendo la inclusión de la enseñanza de RCP en el ámbito escolar^4^, ^5^. La formación de los escolares en reanimación parece contribuir de forma significativa aumentando las tasas de reanimación. Si se empieza a formar a los niños en su edad escolar (Educación Infantil, Educación Primaria y Educación Secundaria Obligatoria) en unos años habrá una sociedad formada en RCP. A partir del año 2021 se plantea ampliar la formación del colectivo escolar incluyendo a los menores de 12 años. Actualmente, el ERC aconseja instruir a este grupo concreto en el «check, call and compress», traducido al castellano como «comprueba, llama y comprime»^2^. En la figura 1A se muestra una propuesta de las competencias por edades para enseñar RCP.

**Figura 1:**
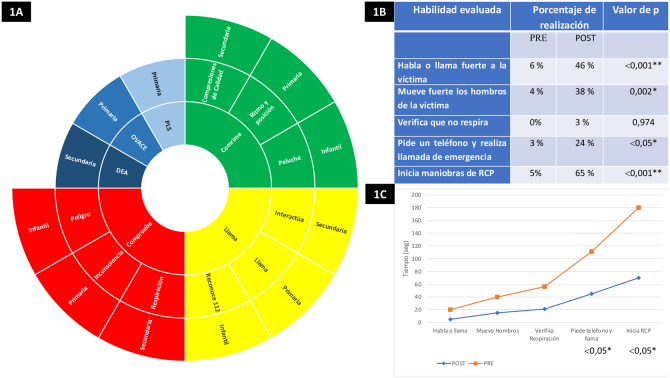
A. Mapa de la distribución de competencias. B. Resultados de la comparación de competencias. C. Resultados de la comparación de tiempos.

Partiendo de esta base y siguiendo las recomendaciones del ERC, se realiza un estudio del efecto de una *Flipped Classroom* o clase invertida en niños de 5 años. Estudio aprobado por el Comité de Ética de la UCAM, número de registro CE022212. Además, se siguieron todos los estándares de confidencialidad e intimidad de los participantes, y la autorización por escrito de los padres y/o tutores. Los participantes y los padres o tutores estaban informados sobre la posibilidad de abandonar el experimento en cualquier momento. Las enfermeras escolares realizaron una valoración inicial, con metodología de aprendizaje basado en problemas.

Posteriormente, se formó a los niños mediante una clase invertida o *Flipped Classroom*, realizada con un videoclip (disponible en el enlace: https://youtu.be/6tiomD-aNu0) siguiendo las últimas recomendaciones del ERC^2^. El videoclip usa los dibujos de «Jacinto y sus Amigos» y la música sigue el ritmo adecuado para hacer bien las compresiones torácicas y contiene una letra que muestra los pasos que debe seguir el niño en una situación de emergencia. El alumno dispuso de los recursos pedagógicos antes de ir al aula donde se hizo otra evaluación.

Para el estudio se analizó una muestra de 122 niños, donde el 47% eran niños y el 53% niñas. Cuatro enfermeras escolares evaluaron al conjunto. En la figura 1A se hace una propuesta de la posible distribución de competencias. En la figura 1B se pueden observar mejoras significativas en 4 competencias y en la figura 1C se advierte una mejora de los tiempos en 2 habilidades en el post. Los resultados del estudio muestran que gracias a la enfermería escolar y mediante una *Flipped Classroom* los niños son capaces de mejorar los conocimientos teóricos y habilidades prácticas en RCP. Además, la propuesta de competencias de la figura 1A puede ser un punto de partida para los diseños de los programas educativos de RCP en la escuela.

## Financiación

Este trabajo ha sido financiado por la Ayuda a la Investigación Ignacio H. Larramendi concedida por Fundación Mapfre, en el año 2022 para el proyecto de investigación PECES (Programa de Educación en Competencias de Emergencia Sanitaria), a la UCAM Universidad Católica de Murcia, al Dr. Manuel Pardo Ríos y su equipo de investigación.

## Conflicto de intereses

Los autores declaran que no hay conflicto de intereses.
